# Opioid Dependent Malingerer with Self-Induced Sepsis

**DOI:** 10.5811/westjem.2016.9.31515

**Published:** 2016-10-20

**Authors:** Kelly A. Kesler, Mark I. Langdorf, Michael J. Burns

**Affiliations:** University of California, Irvine, School of Medicine, Department of Emergency Medicine, Irvine, California

## Abstract

A 21-year-old woman was admitted to the emergency department (ED) with severe sepsis. Both the mechanism of infection and organisms discovered were unusual.

## INTRODUCTION

Physicians learn to identify and treat disease through pattern recognition. But what if the traditional patterns are violated, and the resulting diseases are unknown? How is a bizarre clinical finding rationalized? We present a case in which a societal epidemic, patient subterfuge and microbiologic mystery all intersected to provide a truly unique case report.

There have been few reports of unusual organism bacteremia in intravenous (IV) drug users, and none that we could find in the emergency medicine (EM) literature. A 2016 comprehensive review from *Postgraduate Medical Journal* (from the *BMJ*) of infective endocarditis (IE) lists common culprits as *Staphylococcus aureus*, *Candida albicans* and *Pseudomonas* species, among others, and alludes to saliva contamination among addicts.^1^ A 2012 review in the microbiology/infectious disease (ID) literature lists multiple unusual organisms causing IE,^2^ and a 2016 case report in the ID literature describes a case of persistent *Bacillus cereus* and *Flavimonas bacteremia.*^3^ However, none of these reports identify the organisms found in our patient’s blood. Even though blood culture results return after a patient is admitted from the emergency department (ED), emergency physicians frequently treat IV drug users with malingering behavior, and ferret out unusual causes for life-threatening presentations such as sepsis.

Prescription opioid abuse has risen to epidemic proportions globally. Over the past 2.5 decades, prescriptions for oral narcotics have close to tripled or quadrupled.^4^ The United States is no exception; in 2014, 47,055 individuals in this country died from drug overdose with 18,893 of those related to prescription narcotics alone.^5^ This staggering number represents a 3.4 fold increase since 2000.^5^ In 2008, ED visits for non-medical use of prescribed or over-the-counter medications equaled that of visits related to illicit drugs.^6^ The increase in overdoses from opioid pain medications has risen in conjunction with the increase in narcotic prescriptions.^7^

These opioid analgesics can be taken in many forms; traditionally, the pills are taken by mouth but they can also be crushed and snorted or dissolved and injected intravenously (“mainlining”) or subcutaneously (“skin popping”).

## CASE REPORT

A 21-year-old woman was transported to the ED via ambulance with a complaint of one week of fever, shortness of breath and generalized weakness. She also had a cough productive of green sputum, chest tightness, and redness to both arms at sites of previous peripheral IV insertions and her current midline catheter (peripheral long line to the axillary vein) in her left arm.

She provided a medical history of pulmonary alveolar proteinosis and asthma, and, per chart review, had a history of pseudoseizures, anxiety, and borderline personality. She had been hospitalized multiple times for pulmonary infections, most recently at another hospital twice in the previous week for similar symptoms. She was treated there for both cellulitis and pneumonia and was discharged home on IV daptomycin. She later received a phone call from the other hospital that IV aztreonam would be prescribed in response to a new positive blood culture. She did not recall the names of the bacteria. She self-administered aztreonam and daptomycin just prior to ED arrival. The patient stated that the other hospital inserted a peripheral IV near her left thumb that was removed when her surrounding skin turned red and drained pus. She reported frequent infections, citing cellulitis from multiple small insults, such as IV placements. She also reported anaphylaxis to many antibiotics.

On arrival, her vital signs were blood pressure 99/61 mm/Hg, heart rate 160 beats per minute, respiratory rate 22 respirations per minute, oxygen saturation 100% on 4 liters nasal cannula (NC, chronic home O_2_), and oral temperature 39.5° C. Physical exam showed moderate respiratory distress with the patient only able to speak in short sentences. She was tachycardic without audible murmur, had diffuse rhonchi in all lung fields, and flushed, warm skin. There was redness to her right antecubital fossa at the prior IV insertion site, the dorsum of her left hand, the previous PICC site in her left arm, and also her left antecubital fossa with tenderness over her current home midline IV. In fact, all her current and former upper extremity IV sites appeared red. However, there was no purulent drainage, induration, or areas of fluctuance.

In the ED, the patient received IV fluids and antipyretics. Electrocardiogram revealed sinus tachycardia. Chest radiograph demonstrated hazy bilateral mid and lower lung opacities with low lung volumes with possible subsegmental atelectasis, although consolidation could not be excluded. Serum chemistries measured Na^+^ 128 mmol/L (normal 135–145 mmol/L), K^+^ 2.8 mmol/L (normal 3.4–5.0 mmol/L), HCO_3_^−^ 17 mmol/L (normal 20–29 mmol/L), lactate 0.8 mmol/L, and Mg^++^ 1.3 mg/dL (normal 1.8–2.5mg/dL) and PO_4_^−^ < 1.0 mg/dL (normal 2.5–4.5mg/dL). Complete blood count demonstrated: white blood cell count 2.7 × 10^3^/uL with a normal differential.

The patient was admitted to the internal medicine service on a telemetry bed with a working diagnosis of sepsis from a pulmonary source. As an inpatient, the medicine team obtained her previous medical records. The blood cultures from the other hospital grew *Cronobacter sakazakii* (formerly *Enterobacter sakazakii*) and *Candida parapsilosis*. The current admission blood cultures also grew *C. parapsilosis* as well as *Staphylococcus saccharolyticus*, and the patient was administered micafungin. The patient attempted to block access to other hospital records and only acquiesced if specific documents were not requested (e.g. discharge summary) because they were “wrong.” The ED case manager was notified by the case manager from the patient’s insurance company that the patient was abusive toward staff and left against medical advice (AMA) if she did not receive IV narcotics. She refused lab draws and some antibiotics on the basis of “anaphylaxis” unless she also received IV diphenhydramine and threatened to sign out AMA from the hospital. Her history of pseudoseizures was ultimately confirmed, including resolution of one episode of “convulsions” with IV normal saline. She also maintained that she required continuous NC O_2_, and yet did not desaturate when it was discontinued without her knowledge.

She was transitioned to oral fluconazole and levofloxacin as recommended by the ID consultant. However, the patient refused levofloxacin due to reported anaphylaxis, and she was treated with ciprofloxacin.

Due to her history of line infections, the patient was placed on a 1:1 sitter for suspected line manipulation. The morning of discharge on hospital day 4, the patient was discovered with a syringe filled with partially dissolved hydromorphone that was hidden under her blankets. The sitter reported that the patient had been taking her oral pain medication underneath her blanket and, thus, had not been observed swallowing the pills. The organisms discovered in this patient’s blood, *C. parapsilosis, C. sakazakii*, and *S. saccharolyticus*, have been found in food and on human skin, and in hospital environs. It is likely that the patient was injecting her oral pain medications IV after dissolving them in her mouth, and contaminated her paraphernalia with organisms from her bedding, food, and saliva. She was discharged to home on two weeks of oral ciprofloxacin and fluconazole.

## DISCUSSION

This patient was so debilitated by her substance abuse disorder that she went to extreme measures to potentiate the effects of her oral narcotics by injecting them. The patient caused substantial self-harm and developed recurrent sepsis from pathogens typically found in the mouth, skin and environment. This explained the infection of all her former and current IV lines.

*Cronobacter sakazakii* is a gram negative bacterium that typically does not cause significant infection in adults, but can cause fatal meningitis infections in neonates and young children with underdeveloped immune systems.^8^ This organism is also found in some foods, especially plants, and oral contamination during IV drug use is the likely source of this bacteremia.^9^

*Candida parapsilosis* is a lesser known fungal pathogen in the Candida genus that is commonly found on human skin and was previously unknown as a source of severe infections.^6^ However, it actually can cause significant fungemia and is reported to be the second leading cause of fungemia 10,11 behind *C. albicans* in certain populations. 9,12

*Staphylococcus saccharolyticus* is an anaerobic, coagulase negative pathogen found as native skin flora.^13^ It has been rarely reported to cause nosocomial cases of bacteremia and infectious endocarditis.^14^

## CONCLUSION

The lesson to the astute clinician is to look beyond the usual patterns of disease when faced with atypical presentations. Given the patient’s pseudoseizures, multiple hospitalizations, unusual blood culture results, abusive and obstructive behavior, and deceitful information, it was prudent to investigate her previous records for malingering behavior. Further, the inpatient team astutely assigned a sitter for the patient, who ultimately exposed the root cause of her otherwise puzzling multi-organism bacteremia and sepsis.
